# Attitudes and Willingness to Accept Long-Acting Injections for Patients With Schizophrenia in Beijing: A Cross-Sectional Investigation Based on Samples From the Communities

**DOI:** 10.3389/fpubh.2021.770276

**Published:** 2021-11-25

**Authors:** Junli Zhu, Yun Chen, Wei Lu, Qingzhi Huang, Bin Li, Ying Xu, Rui Xi, Lefan Jin

**Affiliations:** ^1^School of Public Health, Capital Medical University, Beijing, China; ^2^Research Center for Capital Health Management and Policy, Beijing, China; ^3^The National Clinical Research Center for Mental Disorders and Beijing Key Laboratory of Mental Disorders, Beijing Anding Hospital, Capital Medical University, Beijing, China; ^4^Advanced Innovation Center for Human Brain Protection, Capital Medical University, Beijing, China; ^5^Beijing Institute of Mental Health, Beijing, China

**Keywords:** schizophrenia, long-acting injection (LAI), willingness, community patients, clinicians

## Abstract

**Background:** Schizophrenia has brought a serious disease burden to China. Under the background that community rehabilitation has become the mainstream treatment model, the long-acting injection (LAI) can better prevent recurrence. Some districts in Beijing have also issued policies. This article aims to find out patient's current attitudes toward LAI and provide policy suggestions.

**Methods:** Some patients with schizophrenia in the communities are selected, while the survey format is face-to-face conversation. The content of the self-made questionnaire includes patients' willingness and reasons for accepting LAI treatment. Descriptive statistics, *t*-test and F-test are used to process the data from questionnaire surveys.

**Results:** About 10% of respondents have had experience using LAI and the current utilization rate is 2.4%. Respondents' willingness to accept LAI is generally low (only 18.1% are willing). The main reason for willingness is no need to take medication every day, while the main reasons for unwillingness are high cost, fear of injection and lack of understanding.

**Conclusion:** Beijing community patients are not very optimistic about LAI's cognition and willingness. Medication habits play an important role in their medication selection decisions. Intervention such as educate clinicians and patients about LAI and provide free injections to patients can be imposed. The promotion of LAI still has a long way to go.

## Introduction

Schizophrenia is a type of severe mental disorder. The disease has positive psychotic symptoms (hallucinations and delusions) and negative psychotic symptoms [lack of motivation, decreased feeling of pleasure, emotional retardation (1). Its typical characteristics are cognitive and emotional abnormalities, which cause significant damage to the patient's education, occupation, and psycho-social functions (2). The aim of treating schizophrenia is clinical, social and personal remission and prevention of relapses (3). The treatment of schizophrenia mainly starts with oral antipsychotics. At the first episode, 80% of patients treated with oral antipsychotics are likely to be relieved (4). However, nearly three-quarters of patients stop taking medication for various reasons within one and a half years after starting the medication (5), and most patients experience relapse, reduce the quality of life, and increase the risk of causing accidents and suicide (6). It is reported that the risk of recurrence in patients with schizophrenia who discontinue medication is five times that of patients who continue to take medication (7). It would not only increase the financial burden of the patient's family, but also cause huge social and economic losses (8). Global burden of disease research data shows that, globally, the burden of disease caused by mental disorders accounts for 10.5% of the total disease burden. This ratio differs between developed (22.0%) and developing countries (9.0%), and it ranks first in China (14.2%) (9–11).

One type of antipsychotic medicine is long-acting injection (LAI) including first-generation depot antipsychotic (FGDA) and second-generation depot antipsychotic (SGDA) (12). As a new dosage form for the treatment of schizophrenia, LAI has both rapid dissolution and sustained release effects, maintaining a certain blood concentration (13), improving cognitive symptoms, and stabilizing the patient's condition. And a past study has shown that compared with traditional oral medications, LAI therapy can significantly improve medication adherence, help reduce adverse reactions such as extrapyramidal symptoms and malignant syndrome of antipsychotic drugs (14), and then effectively reduce the risk of recurrence and rehospitalization of patients (15, 16). So it is considered an important alternative to oral medications (17). Besides, a health economics evaluation on LAI shows that LAI can also reduce overall medical expenses and resource consumption (18).

The development of LAI is a big boost to “Deinstitutionalization,” making it possible for more patients to return to the community. For a long time in the past, due to the limitations of poor oral medications and poor adherence, hospitalization was the mainstream treatment for schizophrenia. “Deinstitutionalization,” which originated in the late 1950s, is a process led by the American government to reduce the size of mental health specialist medical institutions, to reform the mental health model, and to change ideas (19). It significantly reduces the long-term hospitalization of patients with mental illness and shortens the length of hospital stay on a large scale. At present, it has been widely promoted in European countries. This change has shifted the government's focus from psychiatric hospitals to community rehabilitation institutions. For example, between 1990 and 2002, the number of beds in British psychiatric hospitals decreased by 52%, and government-funded community resettlement housing increased by 40% (20), community mental health received great attention and development, and gradually promoted patient integration into the society. In recent years, China's mental health problems have become increasingly serious. The reported prevalence of severe mental disorders in China rose from 0.36% in 2015 to 0.43% in 2018 (7, 21). However, the allocation of mental health resources in China is far below that of high-income countries (22). Deinstitutionalization has become a reference for solutions to these problems. Since the late 1990s, China has also begun to transform the treatment of mental illness from the traditional mental hospital treatment model to a hospital-community-combined model (19). The model in China has been piloted in 20 provinces or cities, but most of them have not achieved the expected results, and the promotion has been blocked.

In recent years, the prescription rate of LAI in developed countries such as Britain or France in Europe and Singapore in Asia has been maintained at a relatively high level (~20–40%) (23–25). As the most populous country in the world, China's prescription rate for LAI is only 0.66% in 2016, which is even far lower than India (15.8%) (25). Although the second generation of LAI (including injections every January and March) has been launched in China in 2012 and 2018 (26) and has been included in the National Essential Drug List and the Medical Insurance List. However, as of 2020, the utilization rate is still <3% (27). Beginning in 2018, the Chinese government has gradually paid attention to the promotion of LAI. The “Management and Treatment of Severe Mental Disorders (28)” stated: “For patients with poor medication adherence, weak family monitoring capabilities or no monitoring, and risk of causing accidents, LAI treatment is recommended.” Some places have issued LAI implementation policies in their jurisdictions. Some districts in Beijing have also formulated promotion plans. At the beginning of 2019, Chaoyang District in Beijing took the lead in introducing a policy plan to select patients with suitable conditions to vigorously promote LAI (29), and Fengtai District also followed this approach. At present, about 80 patients with high rate of making trouble, poor adherence and weak guardianship accept the free LAI provided by the government. In Tongzhou and Fangshan districts, policies were introduced a little later and the publicity was just completed, but injections have not yet started. The remaining districts have not yet issued policies before September 2020.

The previous literature has shown that the reasons for the low utilization rate of LAI are complex, and the attitude of clinicians and patients is an important factor (30). A study in Switzerland by Jaeger and Rossler pointed out that 75% of psychiatrists would inform patients of different treatment methods (including LAI) and 50% of psychiatrists would recommend LAI treatment for patients after multiple relapses (30). The attitude of patients and their guardians toward LAI is the key to promoting this treatment, the following research can be used as evidence. Although Caroli et al. has shown that more than two-thirds of patients feel better after receiving LAI treatment than before (31), a study by Iyer et al. pointed out that patients who have not used LAI may not be able to correctly understand the meaning of “long-acting” and are worried about the price of treatment (32). A study conducted by Grover et al. from India reported that 78.8% of patients are still willing to choose oral tablets, and only about 5% choose LAI as the first choice of treatment (33). Difficulty in going to the hospital on time for injections, pain and fear of needles, and thinking that it is unnecessary are the reasons why patients often talk about reluctance to accept LAI (33, 34). As mentioned above, China's implementation of the hospital-community-combined model is not very effective. Compared with oral medications, LAI helps stabilize patients' conditions and may help the implementation of this model, thereby alleviating the pressure on mental health resources. However, there is still a lack of research on patients' attitudes toward LAI in China, especially community patients' attitudes. Before vigorously promoting LAI, it is necessary to grasp the attitude and willingness to accept LAI for patients, and to understand the reasons why patients are willing and unwilling to accept, so that corresponding policy interventions can be formulated. This is the focus of this research. This study will focus on filling the gaps in this direction.

## Materials and Methods

### Sample and Data Collection

This study is a cross-sectional survey. The data is collected from the registered patients with schizophrenia in Beijing communities through a questionnaire, which is primarily in the form of face-to-face interviews and with telephone interviews for respondents who have difficulty in moving. The samples are obtained by multi-stage stratified sampling using the following steps: Firstly, the 16 districts of Beijing are divided into three layers according to the special policy status of LAI, including those that have been implemented for more than 1 year (Chaoyang and Fengtai), have just begun to implement (Tongzhou and Fangshan) and have no special policies (the other 12 districts). Secondly, one district is randomly selected from each of the first and second layers, and two districts are selected as representatives from the third layer. Finally, two communities are selected from each district according to the topographical features (Mentougou is a mountainous district, and only one community is selected), and cluster sampling is performed on all registered patients who meet the inclusion and exclusion criteria. Calculate the required sample size according to the sample size formula:


$$\begin{array}{l} \text{n}=\frac{{{\text{u}}_{\text{α}}/2}^{2}\times \pi \left(1-\pi \right)}{\delta^{2}} \end{array}$$

Taking α = 0.05, π = 50%, and δ = 0.05, considering the loss rate of special populations, the sample size is increased by 30%. A total of about 500 samples are drawn from the layers according to the ratio of 4:3:3. There are currently about 50,000 registered patients with schizophrenia in Beijing, and the sampling ratio is about 1%. The survey is conducted anonymously and don't contain any identifiable information. Every interviewee participates voluntarily and signs an informed consent form. This study passed the ethical review of the Medical Ethics Committee of Capital Medical University.

The inclusion criteria for survey subjects of this study are as follows: (1) patients meet the ICD-10 standard and have been diagnosed with schizophrenia. (2) Be at least 18 years old and have a health record in the community. (3) Not currently in hospital, and properly managed in the community. There are also exclusion criteria: (1) Patient has another co-morbid mental illness. (2) Has a high risk of suicide. (3) The physical condition of the patient is insufficient to complete the investigation or refuse to provide valid information. The study lasted for 3 months from August to October 2020. A total of 496 valid data are obtained, including 197 cases in Chaoyang District, 142 cases in Tongzhou District, 96 cases in Changping District, and 61 cases in Mentougou District. The response rate of each community is not <75%.

### Instruments and Statistical Analysis

This study uses self-made questionnaires. To ensure the quality of the questionnaire, two measures are taken: In order to ensure the rationality of the questionnaire, this study uses the expert consultation method to determine the questionnaire items. After about 4 rounds of consultation, the first version of the questionnaire is finalized. The experts in related fields, including four professors of mental health and health economics, three clinicians from psychiatric hospitals, and three psychiatrists from primary health institutions have been consulted. In addition, a preliminary survey based on a small sample has been used to modify instruments.

This questionnaire consists of the following three parts. The first part is the basic information, including age, economic status, occupational status, education level, course of disease, and hospitalization experience. The second part is related to the patient's treatment plan. The investigator would ask the patients about the following information in turn, including medication adherence in the past year, current treatment ways, satisfaction with oral medication treatment and efficacy, whether they have heard of LAI and information acquisition channels, and whether they have ever used LAI in the past. The measurement of medication adherence refers to the questionnaire of Kishimoto et al. (35). Before answering the second part, we educate the respondents who were not aware of LAI on the basic situation of LAI, including the efficacy, mechanism, administration, and price of LAI. The third part is the score of willingness to accept LAI treatment. Using Likert's five-point method, the scores are 1–5 points from very unwilling to very willing. Respondents who score 1–2 points are asked why they are unwilling to accept; respondents who score 4–5 points are asked why they are willing to accept.

In this study, an electronic questionnaire system is used to input and collect data. For measurement data such as willingness scores, the average and standard deviation are used to describe its characteristics; for enumeration data such as basic information and reasons for selection, frequency and percentage are used to describe its distribution. This study mainly use *t*-test and analysis of variance to compare between groups to explore whether there are differences in patients with different characteristics in willingness to accept LAI, SNK-q test and LSD test are used for post-inspection. All processes of statistical analysis are carried out in SPSS 26.0 software.

## Results

### Description of the Basic Characteristics

The demographic characteristics of the overall respondents in this study are shown in Table 1. The 496 respondents in this study include 265 males (53.4%) and 231 females (46.6%). More than one-fifth (20.4%) of the respondents are over 65 years old, followed by those between 41 and 65 years old (58.0%) and ≤ 40 years old (21.6%). 90.5% of respondents are currently unemployed. The highest education level of the respondents is high school, which account for 26.6%; then junior high school account for 50.4%, and primary school account for 23.0%. In term of the course of illness, 16.5% have been sick for over 30 years, followed by those between 11 and 30 years (58.9%) and ≤ 10 years (24.6%). In addition, 63.4% of the respondents have had hospitalization experience.

**Table 1:** Descriptive statistics of basic characteristics.

**Variable**		**Frequency**	**Percentage (%)**
Total sample		496	100.0
Sex	Female	265	53.4
	Male	231	46.6
Age	≤ 40	107	21.6
	41–65	288	58.0
	>65	101	20.4
Occupational status	Unemployed	449	90.5
	Employed	47	9.5
Education level	Primary	114	23.0
	Junior	250	50.4
	High	132	26.6
Course of disease	≤ 10	82	16.5
	11–30	292	58.9
	>30	122	24.6
Hospitalization experience	Never hospitalized	182	36.6
	Only 1 time	157	31.7
	2 times or more	157	31.7

### Description of Issues Related to Treatment

As shown in Table 2, 67.1% of the respondents completely follow the doctor's advice, 19.2% basically follow (Follow 80–120% of the doctor's prescription and occasionally forget), 6.0% don't follow (Uncertain increase or decrease in dosage) and 7.7% refuse treatment (Do not take medicine at all and refuse to take oral medication). 11.5, 21.0, and 67.5% of the respondents are dissatisfied, neutral and satisfied with oral antipsychotics, while 10.7, 21.0, and 68.3% are dissatisfied, neutral and satisfied with oral medication efficacy. 74.6% of the respondents have never heard of LAI, 14.7% have heard of it but never used it, 8.3% had used LAI in the past treatment, only 2.4% are currently using LAI. Among the 126 cases (25.4%) who know about LAI, 73.8% are informed by psychiatrists or nurses, 12.7% are informed by community staffs, 7.2% receive information from wardmates, and 6.3% learn the knowledge from the news media.

**Table 2:** Description of issues related to treatment and reasons to accept LAI treatment.

**Variable (*n* = 496)**	**Frequency**	**Percentage (%)**	**Variable**	**Frequency**	**Percentage (%)**	
					**Within group**	**Total sample**
**Medication adherence**			**Information channels**	126	25.4	
Full compliance	333	67.1	Medical worker	93	73.8	18.8
Basic compliance	95	19.2	Community worker	16	12.7	3.2
Non-compliance	30	6.0	Wardmate	9	7.2	1.8
Refuse medication	38	7.7	News media	8	6.3	1.6
**Oral antipsychotics satisfaction**			**Reasons for willing to accept LAI**	90	18.1	
Dissatisfied	57	11.5	LAI doesn't require every day	64	71.1	12.9
Neutrally	104	21.0	LAI can better prevent recurrence	15	16.7	3.0
Satisfied	335	67.5	Doctors recommend LAI treatment	11	12.2	2.2
**Oral medication efficacy satisfaction**			**Reasons for unwilling to accept LAI**	338	68.1	
Dissatisfied	53	10.7	High cost of treatment	85	25.1	17.1
Neutrally	104	21.0	Unwilling to accept intramuscular injection	84	24.9	16.9
Satisfied	339	68.3	Not comprehending efficacy or side effects	81	24.0	16.3
**Understanding of LAI**			Reluctant to change the medicine	44	13.0	8.9
Never heard of LAI	370	74.6	Refuse to treat because of poor insight	28	8.3	5.7
Heard of but not used	73	14.7	Used before but gave up	16	4.7	3.2
Used in past treatments	41	8.3				
Being treated with LAI	12	2.4				
**Willingness to accept LAI treatment**						
Very unwilling	182	36.7				
Relatively unwilling	156	31.4				
Neutrally	68	13.8				
Relatively willing	57	11.5				
Very willing	33	6.6				

### Willingness and Reasons to Accept LAI Treatment

The willingness to accept LAI and reasons why respondents make such choices are also shown in Table 2. 36.7% are very unwilling to accept LAI, 31.4% are relatively unwilling, 13.8% are neutral, 11.5% are relatively willing, and 6.6% are very willing. Among the 90 respondents with score of 4 or 5, the most respondents think that LAI doesn't require medication every day, which is more convenient (71.1%). The other two reasons are better prevention of recurrence (16.7%) and doctor recommendation (12.2%). Among the other 338 respondents score of 1 or 2, the most unwilling due to high costs (25.1%), followed by intramuscular injection (24.9%), lack of comprehending efficacy or side effects (24.0%). There are also other reasons such as reluctant to change the medicine (13.0%), refuse to take medicine because of poor insight (8.3%), and used before but gave up (4.7%).

As shown in Table 3, the respondents' willingness to accept LAI treatment score is 2.20, which is between “relatively unwilling” and “neutrally.” Different levels of occupational status, educational, oral efficacy satisfaction, understanding of LAI and information channels have statistically significant differences in the willingness to accept LAI. Those who are unemployed are more willing than those who are employed; those with a high level of education are more willing than those with a junior level of education. Other factors, including sex, age, course of disease, hospitalization experience, medication adherence, oral antipsychotics and local policy status, have no significant differences in the willingness to accept LAI.

**Table 3:** Comparison of the willingness to accept LAI treatment.

**Variable**		***χ^2^ ± s***	***t/F***	**Post-inspection**
Total sample		2.20 ± 1.235		
Sex	Female	2.22 ± 1.235	0.299	
	Male	2.18 ± 1.238		
Age	≤ 40	2.36 ± 1.232	1.622	
	41–65	2.19 ± 1.260		
	>65	2.06 ± 1.156		
Occupational status	Unemployed	2.20 ± 1.242	0.768*	“Unemployed” > “Employed”
	Employed	2.15 ± 1.179		
Education level	Primary	2.21 ± 1.208	3.473**	“High” > “Junior”
	Junior	2.08 ± 1.164		
	High	2.42 ± 1.360		
Course of disease	≤ 10	2.30 ± 1.234	1.709	
	11–30	2.24 ± 1.251		
	>30	2.02 ± 1.189		
Hospitalization experience	Never hospitalized	2.28 ± 1.250	1.030	
	Only 1 time	2.09 ± 1.195		
	2 times or more	2.22 ± 1.257		
Medication adherence	Full compliance	2.27 ± 1.279	1.795	
	Basic compliance	2.01 ± 1.116		
	Non-compliance	2.37 ± 1.159		
	Refuse medication	1.95 ± 1.138		
Oral way satisfaction	Dissatisfied	2.00 ± 1.336	0.892	
	Neutrally	2.26 ± 1.231		
	Satisfied	2.21 ± 1.219		
Oral efficacy satisfaction	Dissatisfied	1.81 ± 1.161	3.713**	“Dissatisfied” < “Neutrally” and “Satisfied”
	Neutrally	2.38 ± 1.301		
	Satisfied	2.21 ± 1.216		
Local policy status	Promotion policy (1 year)	2.23 ± 1.334	0.138	
	Promotion policy (started)	2.20 ± 1.146		
	No promotion policy	2.16 ± 1.190		
Understanding of LAI	Never heard of LAI	2.04 ± 1.118	17.771***	“Being treated with LAI” > “Heard of but not used” and “Used in past treatments” > “Never heard of LAI”
	Heard of but not used	2.41 ± 1.352		
	Used in past treatments	2.61 ± 1.358		
	Being treated with LAI	4.33 ± 1.155		
Information channels	Medical worker	2.78 ± 1.458	2.529*	“News media” > “Medical worker” > “Community worker” and “Wardmate”
	Community worker	2.00 ± 1.155		
	Wardmate	2.00 ± 1.000		
	News media	3.25 ± 1.669		

*0.1, **0.05, ***0.01. The bold values is that the variables are statistically significant.

## Discussion

This study has initially grasped the current attitudes of schizophrenic patients in Beijing community toward LAI. The following valuable findings in this study can be emphasized.

The results of this study show that the utilization and awareness rate of LAI among patients with schizophrenia in the Beijing community are very low. Recent epidemiological studies have found that the prescription rates of LAI in clinical settings vary between 20 and 40% in European countries (23, 24, 36), but only 10.7% of respondents have ever used LAI in this study. A study of outpatients has also shown that only about 5% of patients used LAI in China (37), which is lower than that in developed countries. Mace et al.'s investigation has shown that 48% of the community patients in Britain were treated with LAI (38), while Sugawara et al. has reported that 18.2% of outpatients in Japan use LAI (17). Nearly three-quarters of the respondents in this study has never heard of LAI, below the results of Jaeger and Rossler (30) which found that the awareness rate of outpatients is 65.0% in Switzerland. As with previous research results (4, 30), majority of those respondents in this study are aware of LAI from clinicians. This implies that clinicians play a key role in increasing the use of LAI. Although this study does not evaluate the attitude of Beijing clinicians toward LAI, the low awareness rate possibly reflects the negative attitude of clinicians toward LAI. Previous literature has found that clinical psychiatrists had a negative attitude toward LAI (39). Therefore, it is necessary to improve psychiatrists' awareness of the advantages of LAI.

In addition, this study also finds that although patients' attitudes toward LAI are still generally negative after the investigator's science popularization, a sizeable proportion (18.1%) of patients show preference for LAI. The systematic review of Waddell and Taylor in their analysis of 12 articles on LAI use has come to the same conclusion (40). When analyzing the factors affecting the willingness to accept LAI, this study surprisingly finds that the factors that reflect the patient's disease or medication status including course of disease, recent hospitalization experience, medication adherence and the oral way satisfaction have no effect on their attitude. But unlike this study, Grover et al. have found that patients with low medication adherence and hospitalization were less willing (33). This study also shows that there is no significant difference in willingness under different policy states, which may be due to the fact that the policy has just been implemented and has not yet achieved the expected effect. Moreover, patients who are not satisfied with the efficacy of oral medication are less willing to accept LAI. At the same time, it can be found that patients with LAI experience have higher willingness. Heres et al. have found that patients with extensive experience in LAI treatment are much more satisfied in their current treatment than those who are LAI-naive (41). The survey of out-patients of Pereira and Pinto has shown that the great majority of patients (94 and 87%, respectively) receiving either oral medication or LAI would elect to continue with their present form when they were given a free choice (42). This suggests that the patient's medication habits play an important role in their future medication selection decisions.

This study also reports that those who obtained LAI knowledge from the news media have the highest willingness to accept. In China, the government has strict supervision over the news media (43), which is more trusted by patients than other information channels. Stuart pointed out that news media is also an important tool to challenge the prejudice against mental illness and improve education (44). What's more, from the perspective of rational behavior theory (45), obtaining information from the news media is a patient's active search behavior in order to meet their own information needs. Du et al. have proven that the positive news media propaganda can have a significant positive emotional (46). Compared with the media, respondents who obtained information from clinical psychiatrists were less willing to accept LAI, but higher than other channels. Compared to wardmate or community worker, the LAI information delivered by psychiatrists is more professional and positive. Although psychiatrists belong to a relatively trusted group for patients, it is a passive behavior for patients to obtain information from psychiatrists. In addition, studies have shown that doctor-patient contradictions in the field of mental health may be not uncommon (47). It may be prone to low willingness to accept LAI. However, as the most common channel to promote LAI, psychiatrists still play a great role.

This study finds statistically significant differences in LAI acceptance willing by education level. Existing researches have not studied the attitude of patients with LAI from the factor of education level. Although LAI has been clinically used in the United States and European countries for a long time, it is not long in China and unfamiliar to most Chinese patients. Roopun et al. have pointed out that strengthening education can potentially improve treatment acceptance (34). Patients with a high level of education are more capable of accepting new things, so they have a higher willingness to accept LAI in this study. This study also shows that employed patients are less willing to accept LAI than unemployed patients and does not find difference by age and sex. A positive correlation between younger age or male and LAI prescription has been reported in some studies, while others didn't found (48). Two past studies have indicated that some patients may have concerns about stigma and view LAI as reflective of a more severe form of illness (40, 49). Taylor et al. have also showed some patients associate LAI with stigma and coercion (50). In China's government policy on the management of schizophrenia, patients with poor medication adherence, weak family monitoring capabilities or no monitoring, and risk of causing accidents are targets to encourage the implementation of LAI (29) and the same is true in news media reports. For fear of being labeled with more serious illnesses, employed patients show lower willingness, and younger patients do not have a higher willingness in this study. Obviously, the patient's medication habits depend on the psychiatrist's prescription when the disease relapsed in the past, while the introduction of LAI in the early stages of illness will help reduce their association with disease severity and stigma (51).

Regarding the reasons for accepting LAI, about 70% of LAI supporters said that the convenience of not taking medication every day is the reason why they prefer it, which is similar to the results of Wehring et al. (52). However, unwillingness of LAI may be caused by complex reasons in this study. Not comprehending efficacy or side effects is one of the reasons in this study. Moritz et al. have stated that patients often worry about unknown consequences (53). Besides, this study shows that the proportion of patients who are unwilling because of the unacceptable injection way is 24.9%, similar to Grover et al. which showed that the proportions of unwilling to accept LAI due to injection pain and fear of injection were 19.41 and 14.96% (33). In addition, both Meyer and this study report that a considerable number of patients are comfortable with the status quo of treatment and are unwilling to change their medications (54). Due to limited conditions, patients usually can't decide the practical aspects of LAI such as injection site and injection hospital (38, 55). LAI still needs to be improved in terms of those aspects. The use of LAI is also impacted by the high cost particularly for underinsured or uninsured patients, hence making the medicine inaccessible to the patients that need it (56). It is unbearable for those who live on government relief and welfare policies to pay for LAI every month (32). The United States saves the cost of LAI by billing under pharmacy benefits (57), but the cost of LAI is still a considerable limiting factor for use in the United States. We suggest that the government can provide free LAI to patients in poverty to expand policy coverage.

In conclusion, the education of both clinical psychiatrists and patients on LAI knowledge needs to be strengthened urgently. Das et al. have pointed out that the patient's preference can only be exercised when they have information about other available alternative treatment options, while the information about the choices needs to be provided by the psychiatrists (58). And the investigator's popularization of LAI does not make the respondents interested in it like a psychiatrist's introduction (17), which reduces the preference for LAI in this study to a certain extent. Suggestion can be presented that policy can consider optimizing mental health management techniques, improving psychiatrists' knowledge of LAI and strengthening health education for patients at the same time to form a more open relationship and stronger mutual trust.

This study makes significant contributions to the current field of mental health and the treatment of LAI. First of all, unlike most previous clinical studies based on patients in psychiatric hospitals (59, 60), this study is on a natural sample population of community patients. And before confirming the sample, investigators don't know whether respondents have been treated with LAI or oral medication. As some districts in Beijing have already launched LAI community promotion policy, it is urgent to find out the current willingness and attitude of all service targets. In addition, compared with the literature on the attitudes of schizophrenic patients to LAI in developed countries such as France (31), Canada (32), and Italy (61), this study provides empirical evidence under different research backgrounds. China is a country with a low and medium economic development levels, and unlike other developing countries such as India (33), China's political system also has its own characteristics. Finally, this study may be the first to conduct LAI willingness in the context of policy. Then use the result of this study as a basis to change the policy, promote the continuous increase of LAI utilization rate, and promote the update of the entire mental health community management mechanism.

This study also has some limitations. One is that this study uses non-standard self-made questionnaires for on-site surveys. Because there is no standard questionnaire for LAI research, this study can only formulate a questionnaire containing the research information based on the literature (55, 62) and the actual situation in Beijing. The other is due to limited research time and resources, this research is a cross-sectional study, and the strength of causal links is weak. The comparison of LAI preferences of different districts at the same time is not very reliable. A more sophisticated study can be a time series comparison of the same district.

## Conclusion

This study uses a cross-sectional design to conduct a site investigation of schizophrenia patients in Beijing community, and evaluates the attitudes toward LAI and the reasons for their preference. Results have proved that patients as a whole are not willing to accept LAI treatment due to various reasons, and the current utilization rate is low. Policies can be tilted toward the publicity and education of LAI for clinicians and patients. As an important tool to promote the transformation of the mental health community model, LAI helps recover patients to return to society and is worthy of vigorous promotion. In the context of economic globalization, popularizing LAI treatment is not only a more cost-effective way to maintain social stability, but also a big step for China's schizophrenia treatment ways to be in line with the world.

## Data Availability Statement

The raw data supporting the conclusions of this article will be made available by the authors, without undue reservation.

## Ethics Statement

The protocol of this study was approved by the Medical Ethics Committee of Capital Medical University (NO: Z2020SY123). All respondents were voluntary and written informed consent was obtained. All data collection is anonymous.

## Author Contributions

JZ contributed to the conception and design of the study. JZ, YC, WL, QH, BL, YX, RX, and LJ organized the data collection. WL performed the statistical analysis. JZ, YC, and WL wrote sections of the manuscript. All authors contributed to the manuscript revision, read, and approved the submitted version.

## Funding

This study was supported by the Beijing Municipal Natural Science Foundation (Grant no. 9192004) and the National Natural Science Foundation of China (Grant nos. 71573182 and 71974133).

## Conflict of Interest

The authors declare that the research was conducted in the absence of any commercial or financial relationships that could be construed as a potential conflict of interest.

## Publisher's Note

All claims expressed in this article are solely those of the authors and do not necessarily represent those of their affiliated organizations, or those of the publisher, the editors and the reviewers. Any product that may be evaluated in this article, or claim that may be made by its manufacturer, is not guaranteed or endorsed by the publisher.
